# Gelseminum

**Published:** 1877-11-20

**Authors:** I. J. M. Goss


### GELSEMINUM.

BY I. J. M. GOSS, M. A. M. D.

Gelseminum is capable of a wide range of action; it is a polychrest, indeed. It comes in appropriately in those diseases in which the motor portion of the spinal cord is involved. Its power to control the heart in remittent and intermittent fever (in the acme of the fever) is well known. It has a power over certain diseased conditions of the nervous system that cannot be filled by any other remedy. It aids opium, in a marked degree, in subduing pain, and hence, it is one of our best remedies in dysentery, for, it not only acts as an anodyne, but at the same time, it has a specific effect upon that form of inflamation that attacks the mucous membranes. And owing to this property, it is a positive remedy in the early stages of Cartarrh and Gonorrhœa.

I have frequently aborted Gonorrhœa by using Gelseminum internally, in doses of 15 to 20 drops, every three hours; and a wash of Permanganate of Potash for the first one or two days of the attack, when I see the case that early. In sick headache, from determination to the brain, we have no remedy more positive in its curative effects. In Neuralgia of the face and head, it may be alternated with belladonna with the most certain results. In spasmodic affections, it acts with positive certainty. I have often relieved attacks of Asthma with it after Lobelia and other anti-spasmodics had utterly failed. I had a case of Asthma a few days ago, in which I had tried all the various anti spasmodics, as Lobelia, internally, and Nitrite of Amyl, by inhalation, also the vapor of paper steeped in the Nitrate of Potash, and burned in the room, rubbing the chest with chloroform and oil, but all these means failed; finally, I gave the tincture of Gelseminum in doses of 25 drops every three hours, which soon gave relief, producing at the same time quiet and refreshing sleep, which it will generally do, if given in full doses. I use the saturated tincture, made from the green root, for much of it in market is made up from the dry root, and if it be old before it is tinctured, it is worthless.

In cases of obstetrics, where the labor is retarded from a rigid state of the os uteri, there is no remedy that has acted so kindly in my hands. So readily does it overcome the rigidity, and thereby terminate the labor, that some physicians have concluded that it is a valuable parturient, but I have given it to females in the pregnant state, for flux and fever, without any detriment. I am of the opinion that its continued use would cure asthma, provided that the motor system is kept constantly impressed with the remedy, by giving 5 to 10 drops every three or four hours, until that morbid condition is subdued. It is a valuable remedy in cholera-morbus, quieting the system at once, thereby arresting both the vomiting and purging. In that peculiar state of nervous excitement in children from teething, this is one of our best remedies, and if given in time, will prevent spasms, and excessive arterial excitement and fever, so often the results of this condition. It is also a valuable remedy in colliquative diarrhœa of teething children. In tormina and tenesmus of the rectum and bladder, there is no better remedy than gelseminum, for it allays this condition as readily as opium, but does not constipate the bowels. In the early stage of typhoid fever, where there is an excited state of the nervous system, small doses of gelseminum is a valuable remedy, abating the restlessness, and producing sound and refreshing sleep, from which the patient awakes much better. And thus administered, whenever there is an excited condition of the brain and cord, the patient goes through the attack with much less exhaustion than when suffered to do so in this exalted state of the nervous system. Typhoid and typhus fever are disease which materially affect the entire nervous system, and if this element of disease is not looked to, the patient may wear out before the disease can possibly be terminated by any remedial agency. For it is a sad fact, that there has been no specific found for that type of fever as yet, and all that can be done, is to subdue morbid conditions as they occur.

---

Send in your subscriptions for the MEDICAL RECORD for the new year.

